# The legacy of Alex Stacoff for foot and footwear biomechanics research

**DOI:** 10.1186/1757-1146-5-S1-K2

**Published:** 2012-04-10

**Authors:** Peter R Cavanagh

**Affiliations:** 1 Department of Orthopaedics and Sports Medicine, University of Washington, Seattle, WA 98195107, USA

## 

Alex Stacoff was a Swiss biomechanics researcher and a key member of the small group of scientists who founded the International Foot and Ankle Biomechanics Community (i-FAB) in 2007. He was an active participant at the first i-FAB Congress in September 2008, but he died tragically while running, only three weeks later, at the young age of 58 years. The fact that a flourishing i-FAB is now holding its 3^rd^ Congress in Australia is part of Alex’s legacy to the field. But there are other dimensions of his legacy on both the personal and professional levels that I will explore, and hopefully extend, in my presentation. Alex’s professional life was a model of perseverance from which we all can learn. He earned his PhD some 20 years after changing course from an intended career as a schoolteacher. Throughout this time he garnered a reputation for moderation, friendship, mentorship, generosity, and collaborative work that is a model of how research should be conducted. All this was achieved against a backdrop of personal health challenges that would have caused many lesser individuals to abandon professional activities altogether. Alex Stacoff’s professional passion was to understand the biomechanics of the foot and shoe during locomotion. He used both classical and novel methods, the later including bone pin-mounted targets, magnetic resonance imaging kinematics, and fluoroscopy. He expressed an early interest in learning from barefoot running [1] and I will speculate on what he might of thought about the current obsession with this topic. He also used accelerometers attached to the body [2] and, following this lead, I will present new data from our laboratory that demonstrates how skin-mounted accelerometers provide reliable and insightful signals to understand bilateral and intra-subject differences in the foot strike patterns of women distance runners.
